# Polysaccharide from *Atractylodes macrocephala* Koidz. ameliorates DSS-induced colitis in mice by regulating the Th17/Treg cell balance

**DOI:** 10.3389/fimmu.2022.1021695

**Published:** 2022-10-20

**Authors:** Mengjiao Yang, Qianwen Zhang, Reham Taha, Mohammed Ismail Abdelmotalab, Qing Wen, Yuzhu Yuan, Yongrui Zhao, Qingyu Li, Chunyu Liao, Xin Huang, Zhenzhou Jiang, Chenghan Chu, Chunhua Jiao, Lixin Sun

**Affiliations:** ^1^ Jiangsu Center for Pharmacodynamics Research and Evaluation, China Pharmaceutical University, Nanjing, China; ^2^ Department of Gastroenterology, The First Affiliated Hospital of Nanjing Medical University, Nanjing, China

**Keywords:** polysaccharide from *Atractylodes macrocephala* Koidz., ulcerative colitis, transcriptional profile, Th17/Treg cell balance, IL-6/STAT3

## Abstract

*Atractylodes macrocephala* Koidz. is one of the most frequently used traditional Chinese medicines for the treatment of ulcerative colitis (UC). The beneficial effect of polysaccharide from *Atractylodes macrocephala* Koidz. (PAMK) on UC has been reported, while the underlying mechanism and target remain unclear. In this study, we systematically investigated the therapeutic effect and the underlying mechanism of PAMK in UC based on a mouse model of dextran sodium sulfate (DSS)-induced colitis. PAMK treatment (100 mg/kg, 200 mg/kg and 400 mg/kg) significantly ameliorated DSS-induced colitis, manifested as a reduction in weight loss, disease activity index (DAI), colon shortening, spleen index and histological score. Moreover, PAMK treatment inhibited inflammation and improved the integrity of the intestinal barrier in colitis mice. Mechanistically, microarray analysis determined the critical role of the immunoregulatory effect of PAMK in alleviating UC. Flow cytometry analysis further demonstrated that PAMK treatment regulated the balance between T helper (Th) 17 and regulatory T (Treg) cells in the mesenteric lymph nodes (MLN) and spleen in mice with colitis. In addition, PAMK treatment downregulated the expression of IL-6 and suppressed the phosphorylation of STAT3. Together, these data revealed that PAMK treatment alleviated DSS-induced colitis by regulating the Th17/Treg cell balance, which may be dependent on the inhibition of the IL-6/STAT3 signaling pathway. Our study is the first to elucidate that the underlying mechanism by which PAMK treatment alleviates DSS-induced colitis is associated with an improved the Th17/Treg cell balance. Collectively, the study provides evidence for the potential of PAMK to treat UC.

## Introduction

Ulcerative colitis (UC), a subtype of inflammatory bowel disease (IBD), is characterized by chronic and relapsing mucosal inflammation initiating in the rectum and extending upward through part or the entire colon in a continuous fashion (1). The worldwide prevalence of UC has increased in the last few decades. Currently, 5-aminosalicylic acid agents, corticosteroids, immunomodulators, and surgery are the main treatments for UC, which are limited in clinical practice due to common nonadherence, serious adverse effects and heavy financial burden (2). Thus, it is imperative to develop new alternative treatments for UC.

*Atractylodes macrocephala* Koidz. (Baizhu) has been used for improving gastrointestinal function and treating digestive disorders for thousands of years (3), and is currently one of the most frequently used traditional Chinese medicines (TCMs) for the treatment of UC (4). Previous studies demonstrated that combination therapy of a Chinese herbal compound containing Baizhu and mesalazine was more effective in improving the clinical symptoms of UC patients than mesalazine alone (5). Polysaccharide from *Atractylodes macrocephala* Koidz. (PAMK), one of the main components in *Atractylodes macrocephala* Koidz., promoted the proliferation and survival of intestinal epithelial cells *in vitro* (6) and prevented intestinal barrier dysfunction in colitis mice (7). However, the underlying mechanism by which PAMK treatment alleviates colitis remains unclear.

Aberrant immune responses are a hallmark of UC. In UC, large numbers of immune cells are recruited into the inflamed colonic mucous, and then they produce excessive proinflammatory cytokines, resulting in intestinal barrier impairment, gut microbiota dysbiosis and perpetuation of inflammation (8). Immunotherapy drugs, such as anti-IL-12/IL-23 antibodies, have emerged as important treatments for UC by inhibiting the activation of immune cells and the production of their mediators (9). The immunoregulatory effect of PAMK has been determined in previous studies, including promoting the differentiation of Treg cells *in vitro* (10) and enhancing mucosal immunity in the intestine (11). Thus, we investigated the mechanism of PAMK in a mouse model of dextran sodium sulfate (DSS)-induced colitis and hypothesized that the immunoregulatory function plays a key role in its beneficial effect of PAMK on colitis.

## Materials and methods

### Drug

Polysaccharide from *Atractylodes macrocephala* Koidz. (PAMK, purity ≥98.0%) used in our study was purchased from Shanxi Ciyuan Biotechnology Co., Ltd. (Xian, China). PAMK was extracted from *Atractylodis macrocephalae* Rhizoma from Zhejiang Province, China. During the production process, the manufacturer implemented strict process parameters and quality inspection of the intermediates in key processes, such as properties and polysaccharide content. The purity, molecular weight and monosaccharide composition of all final products were investigated. The quality inspection reports offered by the manufacturer showed that there were few differences in the molecular weight and monosaccharide composition between different batches of PAMK, indicating the stable reproducibility of PAMK preparation.

### Reagents

Dextran sulfate sodium (DSS, MW 36000-50000) was obtained from MP Biomedicals (CA, USA). The myeloperoxidase (MPO) assay kit was obtained from Jiancheng Bioengineering Institute (Nanjing, China). Zonula occludens protein 1 (ZO-1) and Occludin antibodies were obtained from Affinity (Liyang, China). FITC anti-CD4, PE anti-Foxp3, and PE anti-IL-17A antibodies were obtained from Biolegend (CA, USA). CD3e antibody, APC anti-CD25 antibody, Foxp3/transcription factor staining buffer set and IC fixation buffer were obtained from eBioscience™ (CA, USA). CD16/CD32 antibody was obtained from BD Biosciences (CA, USA). STAT3 and phospho-STAT3 (p-STAT3) antibodies were obtained from Cell Signaling Technology (MA, USA). The QuantiCyto^®^ Mouse TNF-α enzyme-linked immunosorbent assay (ELISA) kit was obtained from NeoBioscience (Shenzhen, China).

### Characterization of PAMK

#### UV spectra analysis

The UV spectrum of PAMK aqueous solution (40 mg/mL) was recorded with a Nanodrop 2000 system in a region of 220-350 nm to detect free proteins and nucleic acids.

#### Molecular weight analysis

PAMK (11 mg) was dissolved in 1 mL of distilled water and then applied to a gel permeation chromatography system using a PL aquagel-OH MIXED-M column (7.5×300 mm) maintained at a temperature of 40°C. The sample was eluted with 0.1 M NaNO_3_ at a flow rate of 1.0 mL/min. The calibration curve was obtained based on the T-series dextran standards of different molecular weights (T-5, T-10, T-40, T-100, T-500, T-1000 and T-2000).

#### Determination of monosaccharide composition

The monosaccharide composition of PAMK was determined by high-performance liquid chromatography (HPLC). A total of 0.5 mL of 19.4 mg/mL PAMK aqueous solution was hydrolyzed with 0.5 mL of 4 M trifluoroacetic acid (TFA) at 110°C for 2 h. After removal of TFA, the residue was dissolved in 0.5 mL of distilled water, and 200 μL of the solution was acetylated with 120 μL of 0.5 M 1-phenyl-3-methyl-5-pyrazolone (PMP) and 120 μL of 0.3 M sodium hydrate solution in a water bath at 70°C for 2 h. Then, the product was neutralized with 120 μL of 3 M hydrochloric acid and extracted with 2 mL of chloroform thrice with sufficient blending. The aqueous phase was filtered with a 0.22 μm microporous filter and analyzed on a Hypersil BDS-C18 chromatographic column (4.6×150 mm, 5 μm) at 30°C. The mobile phase consisted of phosphate buffer (20 mM, pH 6.7) and acetonitrile as eluents A and B (82:18 v/v). The flow rate was 1.0 mL/min, and the wavelength for UV detection was 250 nm. Eight standard monosaccharides, namely, mannose, rhamnose, glucuronic acid, galacturonic acid, glucose, galactose, arabinose and fucose, were used as references.

### Animals

Specific-pathogen-free (SPF)-grade male C57BL/6J mice weighing 20 ± 2 g were obtained from Shanghai Lingchang Biological Technology Co., Ltd. (Shanghai, China, certificate No. SCXK (hu) 2018-0003). The animals were housed in a specific pathogen-free condition (ambient temperature of 22 ± 2°C, relative humidity of 60 ± 5%, and light-dark cycle of 12 h). All animal experimental protocols were approved by the Ethics Committee of China Pharmaceutical University (No. 2021-09-001).

### Animal experiment

Mice were fed adaptively for 7 days before the experiment and randomly divided into five groups (Control, DSS, DSS+100 mg/kg PAMK, DSS+200 mg/kg PAMK, and DSS+400 mg/kg PAMK). Acute colitis was induced by distilled water containing 3% DSS for 7 days, and the Control group was only given distilled water. From day 8 to day 14, the mice in the five groups received one of the following treatments: Control group (1 ml/100 g normal saline, i.g.), DSS group (1 ml/100 g normal saline, i.g.), DSS+100 mg/kg PAMK (100 mg/kg PAMK, i.g.), DSS+200 mg/kg PAMK (200 mg/kg PAMK, i.g.), and DSS+400 mg/kg PAMK (400 mg/kg PAMK, i.g.). The experimental scheme is shown in **Figure 2A**.

### Evaluation of colitis severity

The body weight, stool consistency and blood in stool were recorded daily during the whole experimental period, and the scoring criteria are shown in **Supplementary Table 1**. The aggregate of all three observations was taken as a disease activity index (DAI). On the 15th day, the mice were sacrificed, and spleen weight and colon length were measured. For histological evaluation, the distal colon tissues were fixed in 10% neutral formaldehyde, dehydrated, and embedded in paraffin. The colon specimens were cut into slices, deparaffinized, stained with hematoxylin and eosin (H&E), and observed under a microscope. The scoring system was based on four independent parameters (**Supplementary Table 2**), and the summation of these scores provided a histopathological score.

### Determination of TNF-α level in plasma

The content of TNF-α in plasma was determined by an ELISA kit according to the manufacturer’s instructions.

### Measurement of MPO activity in colon

The weighed colonic tissue was homogenized with PBS to prepare a 5% homogenate and centrifuged at 12,000 rpm for 5 min. Then, the supernatant was collected, and the MPO activity was determined according to the manufacturer’s protocol.

### Microarray analysis

The Agilent Mouse ceRNA Microarray 2019 (4×180K, Design ID:086242) was used for microarray analysis of the colonic tissues of the Control, DSS and DSS+PAMK (200 mg/kg PAMK, i.g.) groups (n=3). RNA was extracted by a mirVana™ RNA isolation kit following the manufacturer’s instructions. The purity and integrity of total RNA were determined with a NanoDrop ND-2000 and Agilent 2100 bioanalyzer, respectively. RNA was transcribed to cDNA, and then cRNA was synthesized and labeled with Cyanine-3-CTP (Cy3). The labeled cRNA was hybridized onto the microarray. Slides were scanned immediately after washing on the Agilent DNA Microarray Scanner (G2505C), and the scanned images were analyzed with Feature Extraction Software 10.7.1.1 (Agilent) to obtain raw data. Raw data were normalized with the quantile algorithm. Differentially expressed genes (DEGs) and differentially expressed mRNAs (DEMs) were identified through a fold change ≥2 as well as a *P* ≤0.05 calculated with a t test. Gene Ontology (GO) enrichment and Kyoto Encyclopedia of Genes and Genomes (KEGG) enrichment analyses of DEMs were performed to determine the roles of DEMs based on the hypergeometric distribution.

### Quantitative real-time polymerase chain reaction (qRT‒PCR) analysis

Total RNA was extracted from colonic tissues with TRIzol reagent. The concentration and purity were measured by a Nanodrop 2000.Then, cDNA was synthesized with HiScript^®^ Q RT Super Mix, and PCRs were performed with AceQ^®^ qPCR SYBR Green Master Mix as directed by the manufacturer’s protocols. The expression of genes was normalized to the *Actb* gene on the basis of the 2^−ΔΔCT^ algorithm. The primers used in the research are listed in **Supplementary Table 3**.

### Western blotting

The total protein of colonic tissue was extracted using RIPA buffer containing protease inhibitor and phosphatase inhibitor, and the content was determined using a BCA kit. Proteins were separated by SDS‒PAGE and transferred to a PVDF membrane. The membrane was blocked with 5% BSA buffer for one hour at room temperature, followed by incubation with p-STAT3 antibody (1:2000) overnight at 4°C and secondary antibodies for an hour at room temperature. The protein bands were visualized by an automated chemiluminescence Western blot detection system. Then, the same blots were stripped and reprobed with STAT3 antibody (1:1000). Densitometry analysis of bands was performed using ImageJ.

### Flow cytometry analysis

For T helper (Th) 17 cells staining, cells isolated from the spleen and mesenteric lymph nodes (MLN) were stimulated with phorbol-12-myristate-13-acetate (100 ng/mL), ionomycin (1 μg/mL) and brefeldin A (10 μg/mL) at 37°C for 5 h. After blocking with anti-CD16CD32 antibody, the cells were labeled with FITC anti-CD4 antibody. Then, the cells were fixed and permeabilized, followed by intracellular staining with PE anti-IL-17A antibody. Similarly, the surface markers FITC anti-CD4 and APC anti-CD25 antibodies and the intranuclear marker PE anti-Foxp3 antibody were used to label regulatory T (Treg) cells. The data were analyzed using FlowJo 10 software. The proportions of Th17 and Treg cells, and the ratios of Th17/Treg cell in the spleen and MLN in the DSS group and all PAMK treatment groups were normalized to those in the Control group.

### Immunofluorescence staining

The sections of colonic tissues were fixed in 4% paraformaldehyde. For ZO-1 and Occluding staining, the samples were permeabilized with 0.3% Triton X-100 at room temperature for 15 min and blocked with 5% goat serum buffer. For p-STAT3 staining, the samples were permeabilized with methanol at -20°C for 10 min and blocked with 5% goat serum buffer containing 0.3% Triton X-100. Then, the sections were incubated with ZO-1 (1:200), Occludin (1:200) or p-STAT3 (1:100) antibodies overnight at 4°C. The next day, the secondary antibody and 4,6-diamidino-2-phenylindole (DAPI) were used to stain the samples. The stained sections were observed under a fluorescence microscope.

### Statistical analysis

All statistical analyses were performed using GraphPad Prism 8, and the data are presented as the mean ± standard error of the mean (SEM). One-way ANOVA and two-way ANOVA were used for multiple comparisons. A *P <*0.05 was recognized as statistically significant.

## Results

### Structural characterization of PAMK

**Figure 1:**
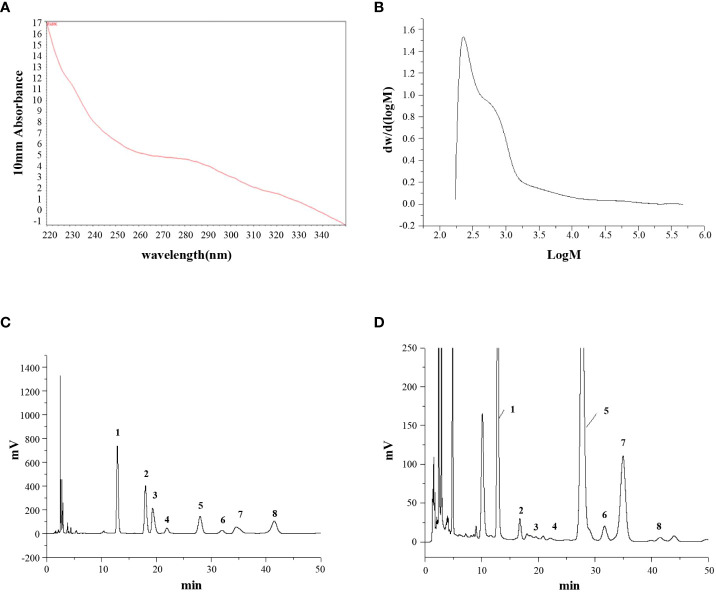
The structural characterization of PAMK. **(A)** UV scan spectrum. **(B)** Gel permeation chromatography spectrum. **(C)** HPLC spectrum of standard monosaccharides. **(D)** HPLC spectrum of PAMK. The peaks numbered 1 to 8 represent mannose, rhamnose, glucuronic acid, galacturonic acid, glucose, galactose, arabinose and fucose, respectively.

The UV scanning spectrum revealed no apparent light absorption at 260 nm and 280 nm, indicating that free proteins and nucleic acids were not present in PAMK (**Figure 1A**). The molecular weight of PAMK was determined to be 2.45×10^3^ Da (**Figure 1B**). Based on the HPLC retention times of standard sugars, the monosaccharide composition of PAMK mainly consisted of mannose, rhamnose, glucuronic acid, galacturonic acid, glucose, galactose, arabinose and fucose in a molar ratio of 7.64, 0.25, 0.17, 0.13, 70.14, 1.00, 6.59, and 0.23 (**Figures 1C, D**).

### PAMK treatment ameliorated DSS-induced colitis

**Figure 2:**
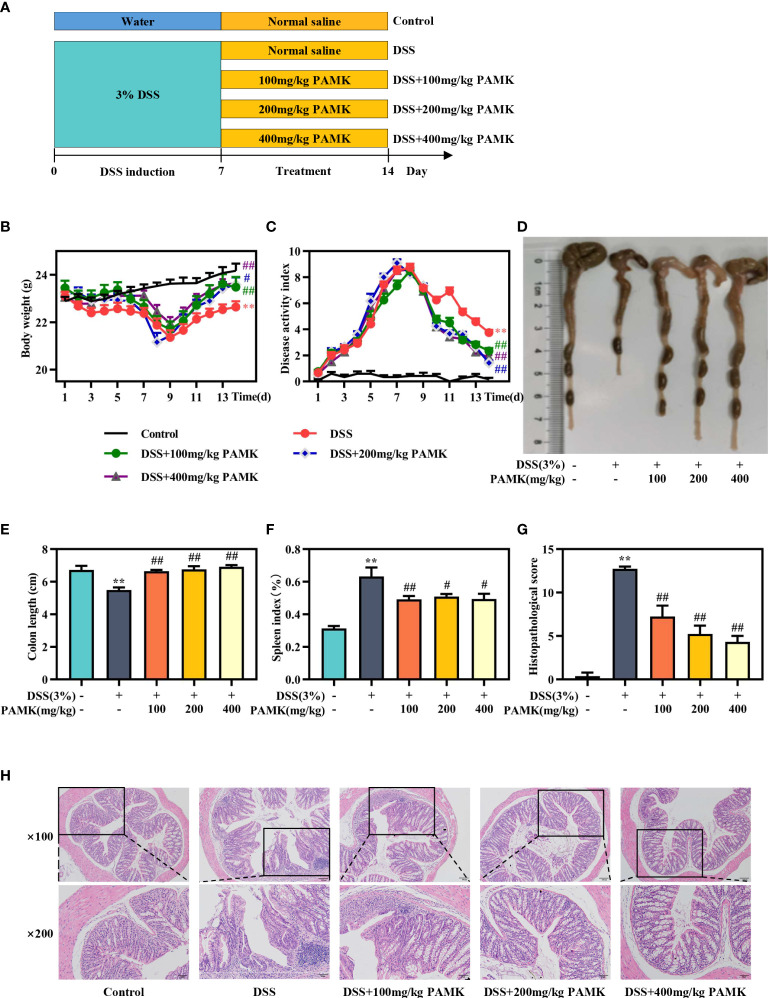
PAMK treatment ameliorated DSS-induced colitis. **(A)** Experimental scheme, **(B)** Daily body weight, **(C)** Daily disease activity index (DAI) scores, **(D)** Representative images of colons, **(E)** Length of colons, **(F)** Spleen index, **(G)** Histological scores of colons, **(H)** Representative images of H&E staining of colon tissue (magnification 100× and 200×). All data are presented as the mean ± SEM (n≥ 6). ^**^ *P <*0.01 vs Control group; ^#^ *P* < 0.05, ^##^ *P* < 0.01 vs DSS group.

The beneficial impacts of PAMK on intestinal functions were examined in a mouse model of DSS-induced colitis. During the experimental process, DSS treatment resulted in weight loss, diarrhea and bloody stool. A significant reduction in weight loss and a marked decrease in the DAI score were observed in all PAMK treatment groups compared with the DSS group (**Figures 2B, C**). PAMK treatment significantly inhibited colon shortening and improved splenomegaly in the mice with colitis (**Figures 2D–F**). H&E staining was performed to systematically evaluate colonic injury. The mice in the DSS group exhibited serious pathological injury, as evidenced by surface epithelium erosion, inflammatory cell infiltration, mucosal ulceration, crypt loss, and goblet cell depletion. These pathological injuries were significantly relieved, and the histological scores significantly decreased after PAMK administration in a dose-dependent manner (**Figures 2G, H**). Together, these results indicated that PAMK treatment significantly ameliorated DSS-induced colitis.

### PAMK treatment inhibited the inflammatory response and improved the intestinal barrier in DSS-induced colitis mice

**Figure 3:**
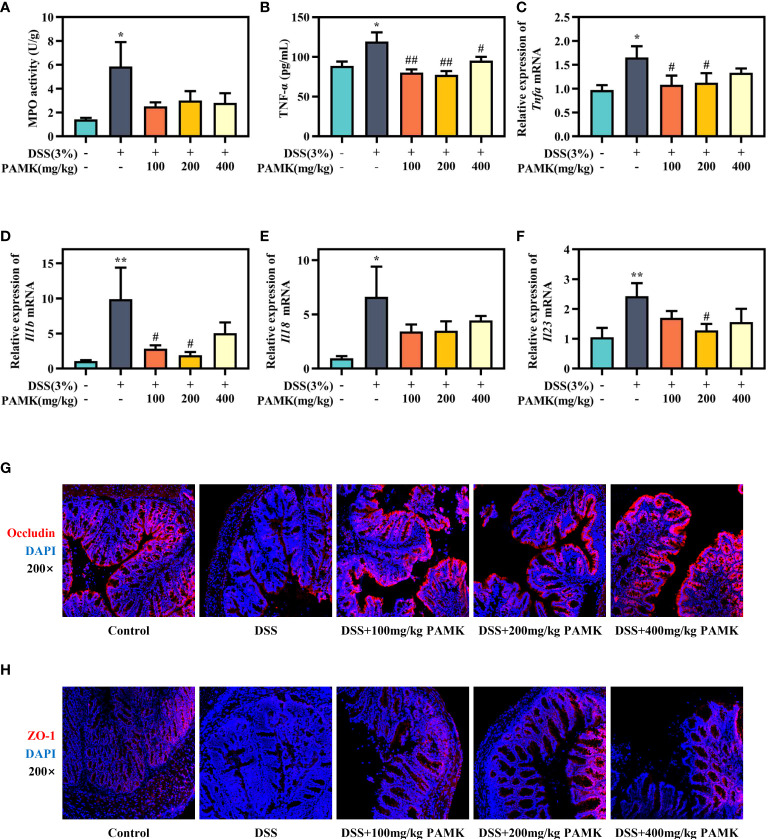
PAMK treatment inhibited the inflammatory response and improved the intestinal barrier in DSS-induced colitis mice. **(A)** MPO activity in the colon. **(B)** The concentration of TNF-α in plasma. The relative expression of *Tnfa***(C)**, *Il1b***(D)**, *Il18***(E)** and*Il23* **(F)** in the colonic tissue. Representative images of immunofluorescence staining of Occludin **(G)** and ZO-1 **(H)** in colonic tissue (magnification 200×). All data are presented as the mean ± SEM (n≥ 6). ^*^ *P <*0.05, ^**^ *P <*0.01 vs Control group; ^#^ *P* < 0.05, ^##^ *P* < 0.01 vs DSS group.

To further assess the effect of PAMK on colitis, MPO activity in colon tissue, an important biomarker of the extent of neutrophil infiltration, was measured, and the data showed that PAMK treatment could decreased MPO activity in colitis mice (**Figure 3A**). UC is known to be caused by overstimulation of proinflammatory cytokines. The level of TNF-α in plasma significantly increased in the DSS group and decreased after PAMK treatment (**Figure 3B**). Similarly, PAMK treatment lowered the expression of proinflammatory cytokines (**Figures 3C–F**), including *Tnfa*, *Il1b*, *Il18* and *Il23*, which have been reported to be increased in UC patients (12, 13). Among the various doses used in the study, 200 mg/kg PAMK treatment showed the strongest anti-inflammatory effect. Impaired intestinal barrier is one of the major pathological features of UC, and tight junction proteins, such as ZO-1 and Occludin, contribute to the integrity of the epithelial barrier (14). The immunofluorescence staining results showed that DSS treatment caused damage to the intestinal barrier in mice, as evidenced by lower expression of ZO-1 and Occludin. PAMK treatment significantly increased the expression of the tight junction proteins (**Figures 3G, H**). The above results indicated that PAMK treatment inhibited inflammation and maintained the integrity of the intestinal barrier in the colon of mice with UC.

### PAMK treatment altered the transcriptional profile in DSS-induced colitis mice

**Figure 4:**
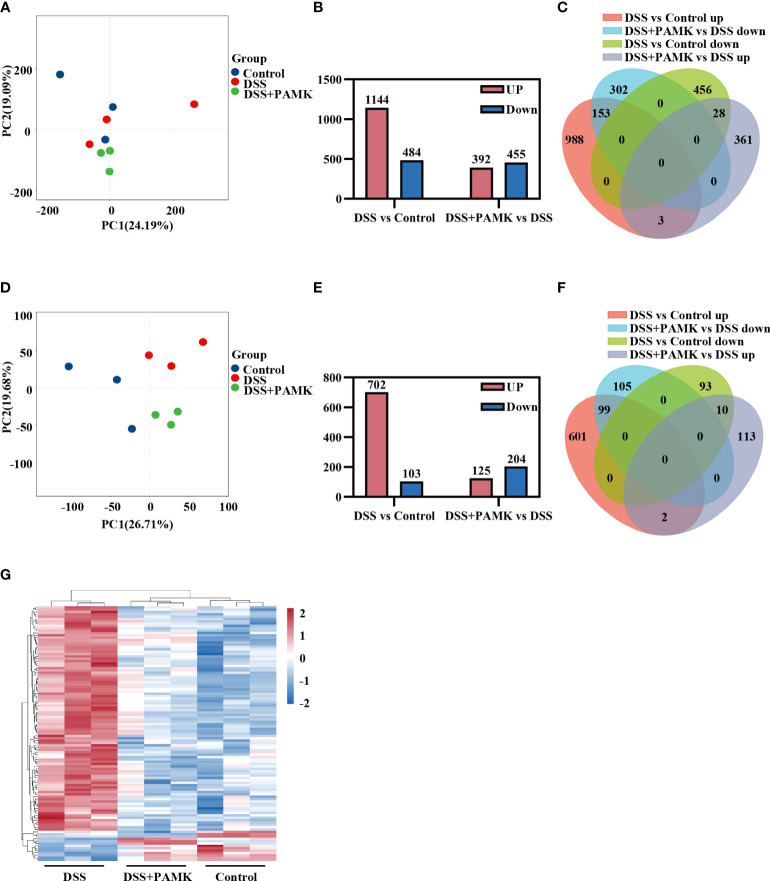
PAMK treatment altered the transcriptional profile in DSS-induced colitis mice. **(A)** Principal component analysis (PCA) of all RNAs from the microarray dataset (n=3). **(B)** The number of differentially expressed genes (DEGs) in the DSS vs Control and DSS+PAMK vs DSS groups (n=3). **(C)** Venn diagram of DEGs in the Control, DSS and DSS+PAMK groups (n=3). **(D)** PCA of mRNAs from the microarray dataset (n=3). **(E)** The number of differentially expressed mRNAs (DEMs) in the DSS vs Control and DSS+PAMK vs DSS groups (n=3). **(F)** Venn diagram of DEMs in the Control, DSS and DSS+PAMK groups (n=3). **(G)** Heatmap of DEMs among the Control, DSS and DSS+PAMK groups (n=3). DEGs and DEMs were identified through a fold change ≥2 as well as a *P* ≤0.05 calculated with a t test.

Microarray analysis was performed to investigate the underlying mechanism for the therapeutic effect of PAMK on colitis. As shown in the principal component analysis (PCA) of DEGs, no obvious separation was observed among the Control, DSS and DSS+PAMK groups (**Figure 4A**). We identified 1144 upregulated DEGs and 484 downregulated DEGs upon comparing the DSS group and Control group. A total of 392 DEGs were upregulated, and 455 DEGs were downregulated in the DSS+PAMK group compared with the DSS group (**Figure 4B**; **Supplementary Excel Sheet**). A Venn diagram showed the DEGs in the comparisons between the DSS vs Control group and the DSS+PAMK vs DSS group (**Figure 4C**; **Supplementary Excel Sheet**). Since there was no significant difference in DEGs between the Control, DSS and DSS+PAMK groups, we focused on the analysis of DEMs. The PCA of DEMs showed that the Control group and DSS group were clearly separated and that the DSS+PAMK group was distinct from the DSS group and tended to be closer to the Control group (**Figure 4D**). We found that 702 DEMs were upregulated and 103 DEMs were downregulated in the DSS group compared with the Control group. A total of 125 DEMs were upregulated and 204 DEMs were downregulated in the DSS+PAMK group compared with the DSS group (**Figure 4E**; **Supplementary Excel Sheet**). PAMK treatment led to the downregulation of 99 DEMs that were significantly increased in the DSS group and resulted in the upregulation of 10 DEMs that were significantly decreased in the DSS group (**Figure 4F**; **Supplementary Excel Sheet**). Thus, 99 upregulated DEMs (DSS vs Control) and 10 downregulated DEMs (DSS vs Control) were considered critical genes reversed by PAMK treatment. The results are presented in the heatmap (**Figure 4G**). These results indicated that PAMK treatment altered the transcriptional profile in the colonic tissues of DSS-induced colitis mice.

### Cluster analysis of DEMs revealed that regulating the Th17/Treg cell balance may be the mechanism of the therapeutic effect of PAMK treatment on DSS-induced colitis

**Figure 5:**
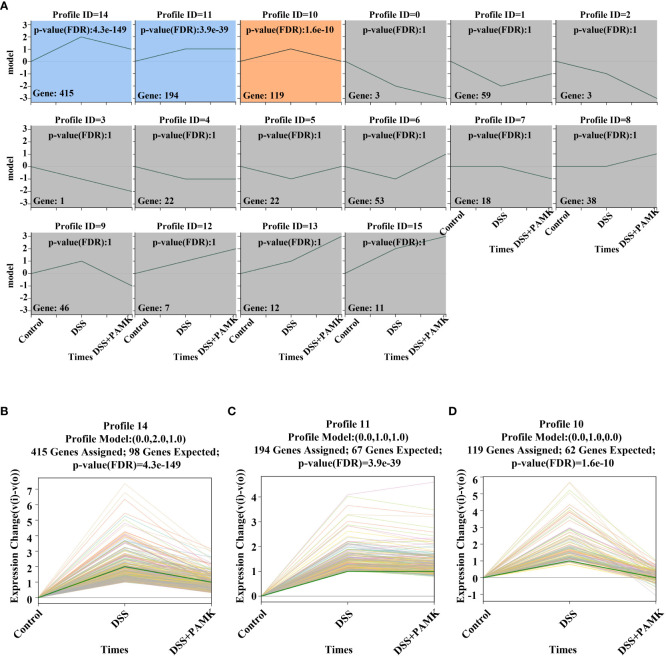
Trend analysis of DEMs. **(A)** Short time-series transcriptomic analysis of DEMs in the Control, DSS and DSS+PAMK groups (n=3). **(B–D)** The statistically significant profiles (*P* < 0.05) of DEMs in the Control, DSS and DSS+PAMK groups (n=3).

**Figure 6:**
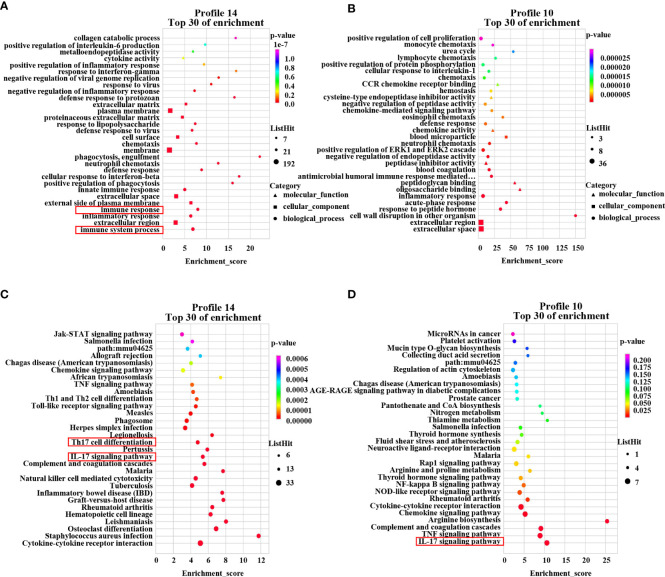
GO enrichment and KEGG pathway enrichment of DEMs. The GO analysis from DEMs of profile 14 **(A)** and profile 10 **(B)** among the Control, DSS and DSS+PAMK groups (n = 3) showed the top 30 enriched biological functions. The KEGG analysis from DEMs of profile 14 **(C)** and profile 10 **(D)** among the Control, DSS and DSS+PAMK groups (n = 3) showed the top 30 enriched signaling pathways.

To further investigate the mechanism of the therapeutic effect of PAMK on DSS-induced colitis in mice, we conducted a trend analysis of the microarray data. The total DEMs were clustered into 16 profiles (from profiles 0 to 15) based on the expression patterns of genes using Short Time-series Expression Miner (STEM) software. Among the 16 profiles, profile 14 (reaching the peak in the DSS group and decreasing in the DSS+PAMK group), profile 11 (reaching the peak in the DSS group and remaining in the DSS+PAMK group) and profile 10 (reaching the peak in the DSS group and returning to the level of the Control group in DSS+PAMK group) were identified with significant (*P*<0.05) expression patterns (**Figure 5**). Profile 14 and profile 10 were the most representative expression patterns. GO enrichment and KEGG enrichment analyses were performed to evaluate the potential functions of DEMs from profile 14 and profile 10. GO analysis, commonly used to analyze the function of genes, showed that the top 30 enriched GO terms of profiles 14 and 10 included immune system process, inflammatory response, immune response, acute-phase response and innate immune response (**Figures 6A, B**). The GO analysis indicated that the beneficial effect of PAMK on DSS-induced colitis may rely on the regulation of the immune response. In addition, the KEGG enrichment of profiles 14 and 10 showed multiple immune-related pathways among the top 30 pathways, including the IL-17 signaling pathway, Th17 cell differentiation, Th1 and Th2 cell differentiation, TNF signaling and the chemokine signaling pathway (**Figures 6C, D**). Therefore, the effect of PAMK on colitis may depend on the immunoregulatory function of PAMK by maintaining Th17/Treg cell homeostasis.

### PAMK treatment regulated the Th17/Treg cell balance in the MLN and spleen in DSS-induced colitis mice

**Figure 7:**
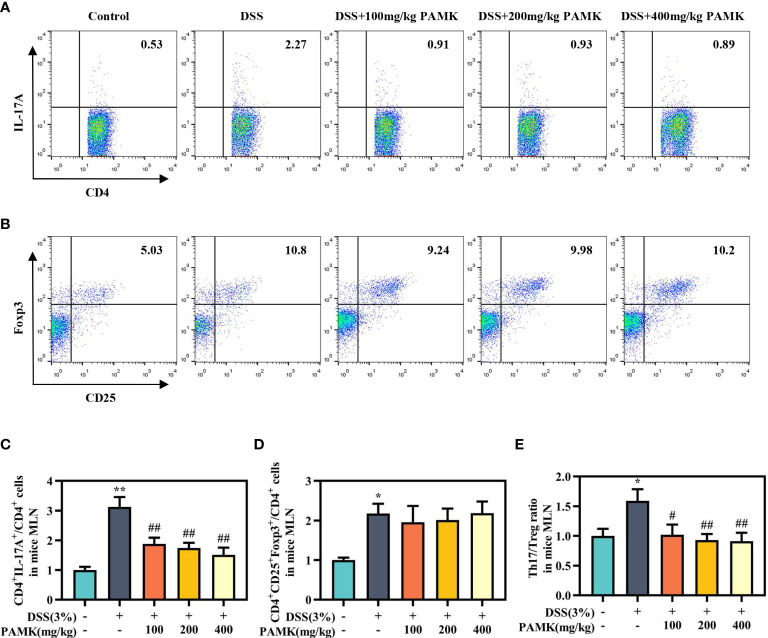
PAMK treatment regulated the Th17/Treg cell balance in the MLN of colitis mice. Flow cytometry of Th17 (CD4^+^IL-17A^+^) cells **(A)** and Treg (CD4^+^CD25^+^Foxp3^+^) cells **(B)** in the MLN. The frequency of Th17 **(C)** and Treg cells **(D)** in the MLN. **(E)** The Th17/Treg ratio in the MLN. The frequency of Th17 and Treg cells and the ratios of Th17/Treg in the DSS group and PAMK treatment groups were normalized to those in the Control group. All data are presented as the mean ± SEM (n≥ 6). ^*^ *P <*0.05, ^**^ *P <*0.01 vs Control group; ^#^ *P* < 0.05, ^##^ *P* < 0.01 vs DSS group.

**Figure 8:**
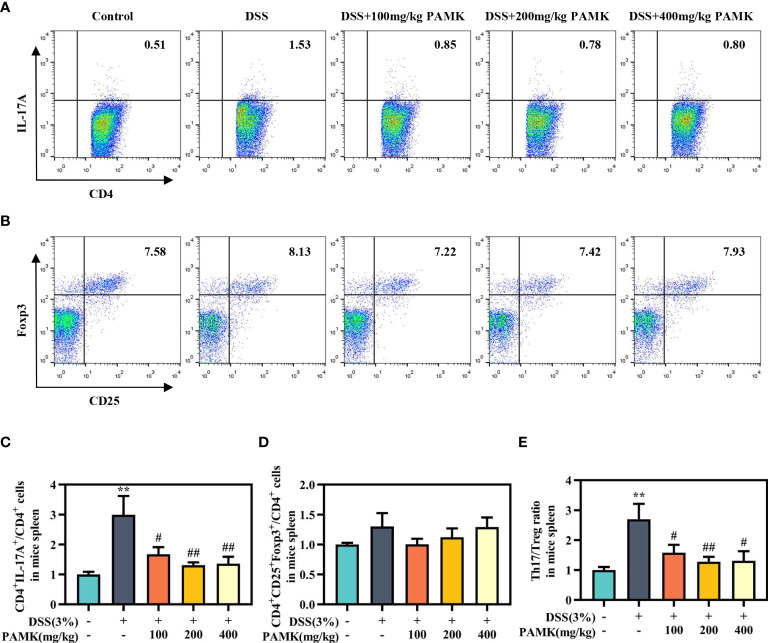
PAMK treatment regulated the Th17/Treg cell balance in the spleen of colitis mice. Flow cytometry of Th17 (CD4^+^IL-17A^+^) cells **(A)** and Treg (CD4^+^CD25^+^Foxp3^+^) cells **(B)** in the spleen. The frequency of Th17 **(C)** and Treg cells **(D)** in the spleen. **(E)** The Th17/Treg ratio in the spleen. The frequency of Th17 and Treg cells and the ratios of Th17/Treg in the DSS group and PAMK treatment groups were normalized to those in the Control group. All data are presented as the mean ± SEM (n≥ 6). ^**^ *P <*0.01 vs Control group; ^#^ *P* < 0.05, ^##^ *P* < 0.01 vs DSS group.

To examine the effect of PAMK on the Th17/Treg cell balance in DSS-induced colitis mice, we measured the proportions of both Th17 cells and Treg cells in the CD4^+^ T cells compartment of the MLN and spleen by flow cytometry. As shown in **Figures 7**, **8**, the proportions of Th17 cells in the MLN and spleen of the DSS group mice were significantly increased compared with those in the Control group mice, and PAMK treatment significantly decreased the proportions of Th17 cells in the MLN and spleen. However, PAMK treatment did not increase the frequency of Treg cells in the MLN and spleen compared with those in the DSS group. The ratios of Th17/Treg cells in the MLN and spleen were shown to decrease after PAMK administration, supporting the role of PAMK in the resumption of the Th17/Treg cell balance in colitis mice.

**Figure 9:**
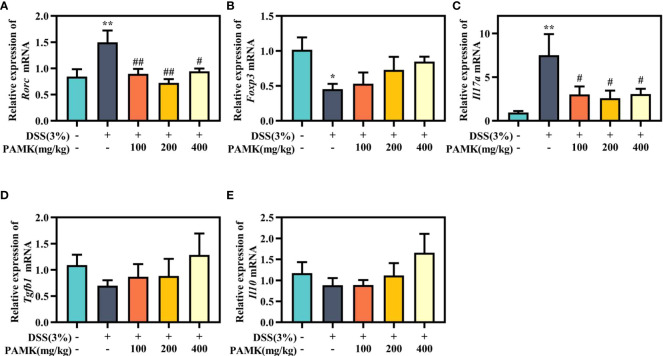
PAMK treatment regulated the levels of Th17- and Treg-specific transcription factors and associated cytokines. The relative expression of *Rorc* **(A)**, *Foxp3* **(B)**, *Il17a***(C)**, *Tgfb1***(D)**, and*Il10***(E)** in the colonic tissue. All data are presented as the mean ± SEM (n≥ 6). ^*^ *P*< 0.05,^**^ *P* < 0.01 vs Control group; ^#^ *P*< 0.05, ^##^ *P* < 0.01 vs DSS group.

Then, Th17- and Treg-specific transcription factors RORγt and Foxp3, as well as the associated cytokines IL-17A, IL-10 and TGF-β1, were analyzed using qRT‒PCR to further investigate the regulatory effect of PAMK on the Th17/Treg cell balance. Significantly increased *Rorc* expression was found in colitis mice in comparison with the Control group, and PAMK treatment significantly decreased the mRNA level of *Rorc* (**Figure 9A**). In addition, the expression of *Foxp3* significantly decreased in colitis and slightly increased in the PAMK treatment groups (**Figure 9B**). The results showed that the expression of Th17- and Treg- associated cytokines was consistent with that of specific transcription factors. IL-17A is secreted specifically by Th17 cells, and TGF-β1 and IL-10 are secreted specifically by Treg cells. The expression level of *Il17a* significantly increased in the colon of the DSS group and decreased after PAMK administration (**Figure 9C**). In contrast, PAMK treatment slightly increased the expression of *Tgfb1* and *Il10* in colitis mice (**Figures 9D, E**). Collectively, these data confirmed the effect of PAMK on maintaining the homeostasis of Th17 and Treg cells in DSS-induced colitis.

### PAMK treatment inhibited the IL-6/STAT3 signaling pathway in DSS-induced colitis mice

**Figure 10:**
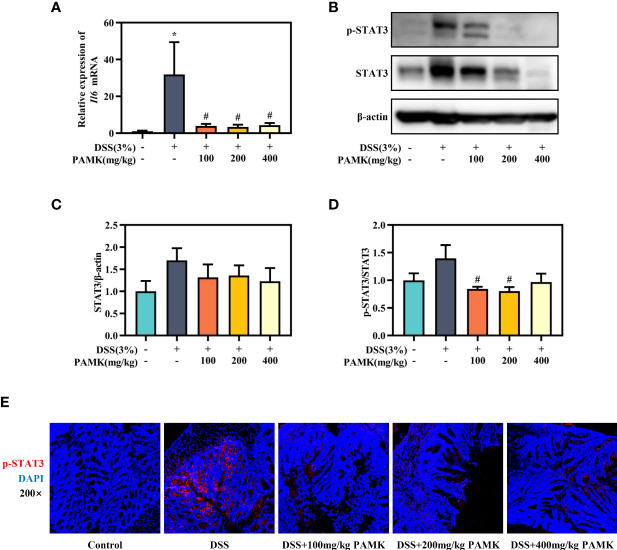
PAMK treatment inhibited the IL-6/STAT3 signaling pathway in DSS-induced colitis mice. **(A)** The relative expression of *Il6* in the colonic tissue. **(B)** Western blot analysis of STAT3 and p-STAT3 expression levels in colonic tissue. **(C)** Densitometric analysis of STAT3 expression. **(D)** Quantitative analysis of p-STAT3/STAT3. **(E)** Representative images of immunofluorescence staining of p-STAT3 in colonic tissue (magnification 200×). All data are presented as the mean ± SEM. ^*^ *P* < 0.05 vs Control group; ^#^ *P* < 0.05 vs DSS group.

The role of the IL-6/STAT3 signaling pathway in the differentiation of Th17 and Treg cells has been reported in multiple studies. Activation of the IL-6/STAT3 signaling pathway drives naïve T cells to differentiate into Th17 cells, resulting in a Th17/Treg cell imbalance (15). Thus, we analyzed the IL-6/STAT3 signaling pathway to explore the mechanism by which PAMK administration modulated the Th17/Treg cell balance. qRT‒PCR results showed that the *Il6* mRNA level in colonic tissue was significantly reduced in the PAMK treatment groups compared to the DSS group (**Figure 10A**). The Western blotting results showed that the level of STAT3 in colonic tissue increased in colitis mice and decreased after PAMK treatment (**Figures 10B, C**). A comparison of p-STAT3/STAT3 in colonic tissues from the DSS and PAMK treatment groups revealed that PAMK treatment decreased the ratio of p-STAT3/STAT3, indicating that PAMK treatment suppressed the activation of STAT3 (**Figure 10D**). The immunofluorescence analysis showed a similar result (**Figure 10E**). Our findings demonstrated that PAMK treatment effectively inhibited the activation of IL-6/STAT3 signaling.

## Discussion

UC, a chronic and recurring intestinal disease, is characterized by abdominal pain, diarrhea and hematochezia, and persistent colitis increases the risk of colorectal cancer (16). 5-aminosalicylic acid (5-ASA) is a first-line therapy for the treatment of UC (17). However, many patients suffer a recurrence after 5-ASA is discontinued, and those patients are recommended to undergo treatment with thiopurines, anti-TNF agents and immunosuppressive therapy despite serious adverse effects (18). Thus, alternative medicines have recently attracted attention in treating UC. Inspiringly, TCMs and polysaccharides from TCMs have become promising alternative and complementary therapies for UC treatment with fewer side effects (19). Jia et al. conducted frequency analysis and found that *Atractylodes macrocephala* Koidz. is one of the most frequently used TCMs in 177 Chinese herbal compound prescriptions used in the clinical treatment of UC (4). Thus, we investigated the effect and the underlying mechanism of PAMK on colitis. This study is the first to elucidate the underlying mechanism of the therapeutic effect of PAMK on colitis and determine that the regulation of the Th17/Treg cell balance of PAMK plays a critical role in alleviating colitis.

The common preclinical murine models of UC include the DSS-induced colitis model, trinitrobenzene sulfonic acid (TNBS)-induced colitis model and IL-10-deficient mouse model (20). Various studies have demonstrated that IL-10 suppresses inflammation in colitis by regulating innate and adaptive immune responses, such as promoting the expansion of Treg cells to suppress the immune response mediated by Th17 cells, which indicates that IL-10-deficient mice were not suitable for the present study (21). TNBS treatment promoted an immune response mediated by Th1 cells, resembling Crohn’s disease but not UC in humans (22). The DSS-induced colitis model is the most widely used murine model of colitis due to its reproducibility, controllability and similarities of pathological and clinical manifestations with UC patients (23). In addition, DSS, as a chemical toxin, causes the damage to the epithelial cells and does not directly activate the adaptive immune system (24), supporting that DSS-induced colitis is the most suitable model for this study. The results showed that PAMK treatment significantly ameliorated DSS-induced colitis manifested as relief of symptoms, inflammation inhibition and intestinal barrier maintenance.

The effects of polysaccharides are intimately associated with their structural properties, especially monosaccharide composition. Numerous studies have demonstrated that the most common monosaccharides contained in polysaccharides from TCMs include glucose, mannose, galactose and arabinose (25). Consistent with the monosaccharide composition of these polysaccharides, the monosaccharide components of PAMK used in our study are mainly glucose, mannose, arabinose and galactose. The effects of monosaccharide treatment on DSS-induced colitis have been determined in previous studies. For instance, both mannose and arabinose treatment alleviated DSS-induced colitis by suppressing the inflammatory response and improving barrier damage (26–28). Galactose is essential for immune system function, and *Astragalus* polysaccharide with higher galactose content showed a more beneficial effect on DSS-induced colitis (29). Therefore, we considered that the protective effect of PAMK against colitis may depend on mannose, arabinose and galactose. However, high glucose treatment exacerbated colitis pathogenesis in mice (30), which may explain why 400 mg/kg PAMK treatment did not exert a more beneficial effect on DSS-induced colitis than 200 mg/kg PAMK treatment. In addition, extensive studies have demonstrated that the polysaccharides from TCMs rich in mannose, arabinose and galactose in colitis, such as polysaccharides from *Inonotus obliquus* (31), *Ganoderma lucidum* (32)and *Dendrobium fimbriatum* Hook (33), alleviate DSS-induced colitis by modulating the immune response. Consistent with these results, our study determined the key role of the immunoregulatory effect of PAMK in ameliorating DSS-induced colitis.

The multifactorial pathophysiology of UC includes environmental factors, disordered intestinal flora, impaired epithelial barrier, genetic predisposition and dysregulated immune responses (34). Among them, a dysregulated immune system accelerates the development of UC (1). In UC, the increasing permeability of the colonic mucosal and epithelial barrier lead to the migration of intestinal flora and triggers the activation of the immune system (35). Stimulated by innate immune cells secreting cytokines, naive CD4^+^ T cells are induced to differentiate into effector CD4^+^ T cells, including Th17 cells and Treg cells (36). The KEEG enrichment analysis revealed that the effect of PAMK on colitis was associated to the Th17/Treg cell balance. Under physiological conditions, Th17 cells protect the host against infection and mediate the immune response by secreting inflammatory cytokines. Treg cells maintain immune tolerance and prevent an excessive immune response by secreting anti-inflammatory cytokines (37). In UC patients, Th17 cells infiltrate the gastrointestinal mucosa and produce excessive inflammatory cytokines, such as IL-17A, initiating a more intense inflammatory response that Treg cells are not able to tolerate (38). Thus, Th17/Treg cell imbalance is a crucial factor in the occurrence and development of UC, and targeting the regulation of the Th17/Treg cell balance has been a promising strategy for treating UC (15). In our study, PAMK treatment significantly decreased the frequency of Th17 cells in the spleen and MLN, and the expression of *Il17a* in colonic tissue in colitis mice. In UC patients, the proportion of Treg cells is decreased in peripheral blood, while it is increased in the inflamed mucosa of the colon, which may be attributed to the active recruitment of Treg cells in inflamed areas to maintain immune tolerance and inhibit the inflammation (39). Consistently, our study also showed that the frequency of Treg cells in the MLN and spleen increased in colitis mice. However, the instability of Foxp3^+^ Treg cells in colitis has been identified, and multiple studies have demonstrated that the expression of Foxp3 in Treg cells isolated from inflammatory sites decreased, indicating a loss of Foxp3 expression (40), which may explain why the proportions of Treg cells increased in colitis mice, while the expression of *Foxp3*, *Il10* and *Tgfb1* decreased. The upregulation of *Foxp3*, *Il10* and *Tgfb1* expression in the PAMK treatment groups indicated that PAMK treatment enhanced the activation of Treg cells. Our study indicated that PAMK treatment alleviated DSS-induced colitis by regulating the Th17/Treg cell balance.

Finally, we explored the pathways that promoted differentiation and enhanced the secretion of Th17 cells. Genome-wide association studies have determined that STAT3 is linked to UC susceptibility. STAT3 is expressed in mucosal immune cells, and STAT3 activation enhances the inflammatory response in the intestine (41). IL-6 initiates the phosphorylation of STAT3 by binding the membrane-bound IL-6 receptor (42), and p-STAT3 is an essential mediator of the Th17/Treg cell balance and upregulates the expression of Th17-specific genes, such as IL-17A (38). Thus, the IL-6/STAT3 signaling pathway plays a pivotal role in the differentiation of Th17 cells. In UC, the phosphorylated STAT3 levels were significantly increased in actively inflamed colons (43, 44). Target blockade of the IL-6/STAT3 signaling pathway, such as blockade with IL-6 antagonists, JAK inhibitors and direct STAT3 inhibitors, can effectively prevent and treat UC (45). Many polysaccharides from TCMs have been demonstrated to inhibit the IL-6/STAT3 pathway in DSS-induced colitis mice (46, 47). Our results showed that the regulatory effect of PAMK on the Th17/Treg balance in colitis may depend on the inhibition of the IL-6/STAT3 pathway.

## Conclusion

The study evaluated the effect of PAMK on colitis and determined the underlying mechanism based on a mouse model of DSS-induced colitis. PAMK improved colitis symptoms, alleviated pathological injury, inhibited the inflammatory response and improved the intestinal barrier in colitis mice. In addition, the beneficial effect of PAMK on colitis depended upon the immunoregulatory effect. Our results suggested that PAMK treatment regulated the Th17/Treg cell balance to attenuate DSS-induced colitis in mice, which may be dependent on the inhibition of the IL-6/STAT3 signaling pathway. These findings provide further evidence for the potential of PAMK for treating UC.

## Data availability statement

The datasets presented in this study can be found in online repositories. The names of the repository/repositories and accession number(s) can be found below: https://www.ncbi.nlm.nih.gov/geo/, GSE211359.

## Ethics statement

The animal study was reviewed and approved by Ethics Committee of China Pharmaceutical University.

## Author contributions

LS and CJ conceived and designed the study. QZ and MY performed the experiments. MA, QW, YY and YZ helped with several experiment procedures. QL, CL, ZJ, XH and CC analyzed the data. MY drafted the paper. LS and RT revised the paper. All authors contributed to the article and approved the submitted version.

## Funding

This work was supported by the National Natural Science Foundation of China (82074115, 81873084 and 82174072).

## Acknowledgments

We thank the Laboratory of Active Components and Pharmacodynamics of Natural Medicines of China Pharmaceutical University and OE Biotechnology Co., Ltd. for laboratory assistance.

## Conflict of interest

The authors declare that the research was conducted in the absence of any commercial or financial relationships that could be construed as a potential conflict of interest.

## Publisher’s note

All claims expressed in this article are solely those of the authors and do not necessarily represent those of their affiliated organizations, or those of the publisher, the editors and the reviewers. Any product that may be evaluated in this article, or claim that may be made by its manufacturer, is not guaranteed or endorsed by the publisher.
