# Supplementary material for: Polysaccharide from Atractylodes macrocephala Koidz. ameliorates DSS-induced colitis in mice by regulating the Th17/Treg cell balance
Source: Front Immunol. 2022 Oct 20;13:1021695. doi: 10.3389/fimmu.2022.1021695 (PMC9630481; doi:10.3389/fimmu.2022.1021695)
Supplement: Supplementary file 1 [file Table_1.docx]

**Supplementary Table 1. Overview of the criteria used for the scores**

| **Score value** | **Body weight loss** | **Stool consistency** | **Blood in stool** |
| --- | --- | --- | --- |
| 0 | ≤1% | Normal stool | Colorless in 2 minutes |
| 1 | 1-5% | Softer stool | Color changes from light green to green within 10 seconds |
| 2 | 5-10% | Extremely softer but formed stool | Color changes from light green to  blue‒brown |
| 3 | 10-15% | Unformed stool | Color changes from blue‒brown to  dark-brown |
| 4 | >15% | Watery stool | Color immediately becomes black‒brown |
| 5 | - | - | Macroscopic bloody stool |
| 6 | - | - | Macroscopic blood in anus and rectum |

The scoring criteria of blood in stool is offered by the manufacturer (Shanghai Yuanye Biotechnology Co., Ltd.).

**Supplementary Table 2. Histological score criteria**

|  | **Score value** | **Definition** |
| --- | --- | --- |
| Extent of inflammation | 0 | None |
|  | 1 | Slight |
|  | 2 | Mild |
|  | 3 | Moderate |
|  | 4 | Severe |
| Depth of injury | 0 | None |
|  | 1 | Mucosa |
|  | 2 | Submucosa |
|  | 3 | Muscular layer |
|  | 4 | Serosal layer |
| Crypt damage | 0 | None |
|  | 1 | Small proportion of crypt/epithelium damaged. |
|  | 2 | Moderate proportion of crypt/epithelium damaged. |
|  | 3 | Majority of crypt/epithelium damaged. |
|  | 4 | All crypt/epithelium damaged. |
| Diseased region | 0 | None |
|  | 1 | 1-25% |
|  | 2 | 26-50% |
|  | 3 | 51-75% |
|  | 4 | 76-100 |

**Supplementary Table 3. List of primers**

| **Gene** | **Primers** |
| --- | --- |
| *Tnfa* | Forward: CCCTCACACTCAGATCATCTTCT |
|  | Reverse: GCTACGACGTGGGCTACAG |
| *Il1b* | Forward: CTGTGTCTTTCCCGTGGACC |
|  | Reverse: CAGCTCATATGGGTCCGACA |
| *Il18* | Forward: ACTGTACAACCGCAGTAATACGG |
|  | Reverse: AGTGAACATTACAGATTTATCCC |
| *Il23* | Forward: AGCGGGACATATGAATCTACTAAGAGA |
|  | Reverse: GTCCTAGTAGGGAGGTGTGAAGTTG |
| *Foxp3* | Forward: CACCCAGGAAAGACAGCAACC |
|  | Reverse: CTCGAAGACCTTCTCACAACCA |
| *Rorc* | Forward: ACAAATTGAAGTGATCCCTTGC |
|  | Reverse: GGAGTAGGCCACATTACACTG |
| *Il6* | Forward: TCTGAAGGACTCTGGCTTTG |
|  | Reverse: GATGGATGCTACCAAACTGGA |
| *Il17a* | Forward: TCAGCGTGTCCAAACACTGAG |
|  | Reverse: CGCCAAGGGAGTTAAAGACTT |
| *Il10* | Forward: GGTTGCCAAGCCTTATCG |
|  | Reverse: TCTTCACCTGCTCCACTG |
| *Tgfb1* | Forward: AGCTGCGCTTGCAGAGATTA |
|  | Reverse: AGCCCTGTATTCCGTCTCCT |
| *Actb* | Forward: CATTGCTGACAGGATGCAGAAGG |
|  | Reverse: TGCTGGAAGGTGGACAGTGAGG |
